# Development of a Genetic System for *Marinobacter atlanticus* CP1 (*sp. nov.*), a Wax Ester Producing Strain Isolated From an Autotrophic Biocathode

**DOI:** 10.3389/fmicb.2018.03176

**Published:** 2018-12-21

**Authors:** Lina J. Bird, Zheng Wang, Anthony P. Malanoski, Elizabeth L. Onderko, Brandy J. Johnson, Martin H. Moore, Daniel A. Phillips, Brandon J. Chu, J. Fitzpatrick Doyle, Brian J. Eddie, Sarah M. Glaven

**Affiliations:** ^1^National Research Council, Washington, DC, United States; ^2^Center for Biomolecular Science and Engineering, United States Naval Research Laboratory, Washington, DC, United States; ^3^American Society For Engineering Education, Washington, DC, United States; ^4^Fischell Department of Bioengineering, University of Maryland, College Park, College Park, MD, United States; ^5^Sarah Lawrence College, Bronxville, NY, United States

**Keywords:** *Marinobacter*, wax ester synthesis, biocathode, genetic system, mutagenesis

## Abstract

Here, we report on the development of a genetic system for *Marinobacter* sp. strain CP1, previously isolated from the Biocathode MCL community and shown to oxidize iron and grow as a cathodic biofilm. Sequence analysis of the small and large subunits of the 16S rRNA gene of CP1, as well as comparison of select conserved proteins, indicate that it is most closely related to *Marinobacter adhaerens* HP15 and *Marinobacter* sp. ES.042. *In silico* DNA–DNA hybridization using the genome-to-genome distance calculator (GGDC) predicts CP1 to be a new species of *Marinobacter* described here as *Marinobacter atlanticus*. CP1 is competent for transformation with plasmid DNA using conjugation with *Escherichia coli* donor strain WM3064 and constitutive expression of green fluorescent protein (GFP) is stable in the absence of antibiotic selection. Targeted double deletion mutagenesis of homologs for the *M. aquaeoli* fatty acyl-CoA reductase (*acrB*) and fatty aldehyde reductase (*farA*) genes resulted in a loss of production of wax esters; however, single deletion mutants for either gene resulted in an increase in total wax esters recovered. Genetic tools presented here for CP1 will enable further exploration of wax ester synthesis for biotechnological applications, as well as furthering our efforts to understand the role of CP1 within the Biocathode MCL community.

## Introduction

*Marinobacter* spp. have been isolated from a wide range of ecological niches, including subsurface environments (Bonis and Gralnick, 2015; Leslie et al., 2015; Evans et al., 2018) and hydrocarbon contaminated water (Gauthier et al., 1992), and participate in important environmental processes such as biogeochemical cycling as reviewed by Singer et al. (2011) and Handley and Lloyd (2013). *Marinobacter* has been called a “biogeochemical opportunitroph” (Singer et al., 2011) due to the metabolic flexibility of this genera and apparent ability to use a variety of carbon sources as well as to interact with metals (Edwards et al., 2003; Handley et al., 2009; Bonis and Gralnick, 2015; Wang et al., 2015b). *Marinobacter* spp. are known to produce wax esters (Barney et al., 2012; Lenneman et al., 2013), and wax ester synthesis pathways have been studied for their potential use in biotechnology applications including in the medical, food, and cosmetic industries, as well as in high-grade lubricants (Wahlen et al., 2009).

More recently, *Marinobacter* spp. have also been found in the microbial communities that colonize cathode electrodes of bioelectrochemical systems (BES) (Rowe et al., 2014; Debuy et al., 2015; Wang et al., 2015b; Stepanov et al., 2016), suggesting the possibility for extracellular electron transfer (EET) with a solid surface. As previously reported by our group, *Marinobacter* sp. CP1 is a dominant member of the Biocathode MCL community, a stable microbial community enriched from seawater from the Atlantic ocean which grows and produces current on a high potential (310 vs. SHE) electrode with no added carbon. When grown as a pure culture, *Marinobater* sp. CP1 is able to interact with an electrode when supplemented with organic carbon (Leary et al., 2015; Wang et al., 2015b). Using metagenomic and 16S rRNA sequencing, it was shown that CP1 was the only member of the MCL community whose relative abundance increased with reduced cathodic electron transfer (Malanoski et al., 2018). Although the role of CP1 in the MCL community is unknown, this observation suggests some critical interaction occurs between CP1 and the electrode surface, and/or the dominant electroautotroph, “*Ca.* Tenderia electrophaga” (Eddie et al., 2016). Previous reports of *Marinobacter adhaerens* HP15 (Sonnenschein et al., 2011), *M. aquaeolei* VT8 (Lenneman et al., 2013), and *M. subterrani* (Bonis and Gralnick, 2015) have shown that this genus is genetically tractable; therefore, we developed a genetic system for CP1 in order to support efforts to understand its role in the MCL community. Also of interest is CP1’s capacity for carbon storage through wax ester synthesis. As wax esters have long been of interest for their use as a feedstock for many products – including cosmetics, lubricants, and other wax-containing products – (Ishige et al., 2003), the presence of the wax ester synthesis pathway suggests a potential for use of *Marinobacter* CP1 as a host organism for synthetic biology applications. Here, we describe optimal growth conditions for CP1, show that CP1 is amenable to genetic transformation by conjugation, and demonstrate mutagenesis of a putative wax ester synthesis pathway *via* markerless deletion of genes homologous to the *M. aquaeolei* VT8 fatty acyl-CoA reductase (*acrB*) and fatty aldehyde reductase (*farA*). We also provide evidence that *Marinobacter* sp. strain CP1 is taxonomically distinct from *M. adhaerens* and propose classification as a new species.

## Materials and Methods

### Phylogeny

The phylogenetic position of *Marinobacter* sp. strain CP1 was determined using both the 16S and 23S rRNA gene (16S and 23S), as well as conserved protein sequences. Strains within the genus *Marinobacter* with complete genome sequences as well as members of the family *Alteromonadaceae* that had both 16S and 23S regions sequences in the database were included in phylogenetic trees. All 16S and 23S sequences were obtained from the Integrated Microbial Genomes (IMG) database^1^ and the National Center for Biotechnology Information (NCBI^2^). 16S and 23S sequences were aligned using the SILVA Incremental Aligner (SINA), which takes into account secondary and tertiary structure (Pruesse et al., 2012). After deleting gap only sites from these alignments, the program Clustal Omega v. 1.2.4 (Sievers et al., 2011) was used to generate a multiple sequence alignment of each region. The alignments were then used to reconstruct phylogenetic trees using MEGA6 (Tamura et al., 2013). For all phylogenetic trees, the “Model Selection” tool was used to identify the evolutionary model with the lowest Bayesian information criterion score that was compatible with the type of analysis. For both the 16S and 23S sequences, this was the general time reversible (GTR) model assuming a gamma distribution of changes between sites (Nei and Kumar, 2000) for maximum-likelihood (ML) analysis. All positions with less than 95% site coverage were eliminated. That is, fewer than 5% alignment gaps, missing data, and ambiguous bases were allowed at any position.

The CheckM database v.1.0.4 (Parks et al., 2015) was previously used to generate a selection of conserved genes from isolate genomes of Biocathode MCL [see Table S6 in Malanoski et al. (2018)] and were used here for phylogenetic comparison of CP1 to near complete genomes of *Marinobacter* spp. and members of the family *Alteromonadaceae*. The resulting sequences were aligned using Clustal Omega, which was then used to reconstruct an ML phylogenetic tree, using the La Gascuel model with 500 bootstrap replications (Le et al., 2008). In addition, all available fully complete genomes of *Marinobacter* spp. were submitted to the genome-to-genome distance calculator (GGDC) website^3^ (Meier-Kolthoff et al., 2013) to determine a digital DNA–DNA hybridization (dDDH) to CP1. Sub-strains of the same species reported in NCBI were also compared to themselves as well as CP1. Predicted DDH values and values that cover the model confidence interval (CI) are reported. BLASTP (NCBI) was used to compare CP1 to its closest phylogenetic match, *M. adhaerens*, to determine the closest matching proteins [expect value (*E*) 0.000001]. KEGG pathway entries for both organisms were retrieved and compared to identify unique pathways between the two species.

### Bacterial Strains, Plasmids, and Medium

#### Development of BB Medium

As described in Wang et al. (Singer et al., 2011), *Marinobacter* CP1 was isolated by plating cell resuspended from the electron on Difco Marine Broth (MB) plates, and restreaking individual colonies for purity. Once isolated, the strain was regularly cultivated from a glycerol freezer stock at 30°C with shaking in rich medium that is either Difco MB 2216 (Becton Dickinson, Franklin Lakes, NJ, United States), or one half lysogeny broth (LB) (Luria and Burrous, 1957) and one half MB, which is referred to here as BB medium. For 1 L of BB medium 18.7 g of Difco MB 2216, 5.0 g of tryptone, 2.5 g of yeast extract, and 5.0 g of sodium chloride. For agar plates, 15.0 g of agar were added to the medium prior to autoclaving.

#### Growth in Defined Minimal Artificial Seawater Medium

When cultivated in defined medium, *Marinobacter* CP1 was grown at 30°C with shaking in minimal artificial seawater (ASW) medium for neutrophilic iron-oxidizing bacteria (Emerson and Floyd, 2005) with a lower concentration of CaCl_2_ than previously reported for Biocathode MCL to support planktonic growth: 27.50 g NaCl, 3.80 g MgCl_2_⋅6H_2_O, 6.78 g MgSO_4_⋅H_2_O, 0.72 g KCl, 0.62 g NaHCO_3_, 0.05 g CaCl_2_⋅2H_2_O, 1.00 g NH_4_Cl, 0.05 g K_2_HPO_4_, and 1 mL Wolfe’s Trace Mineral Solution per liter supplemented with 7 g sodium succinate dibasic hexahydrate as a carbon source. The medium is brought to a final pH of 6.5–6.8 with CO_2_.

#### Growth Assays

When investigating which carbon sources supported growth of CP1, cells were cultivated at 30°C with shaking in defined ASW medium supplemented with those carbon sources listed in Table 1. Each carbon source was used in place of the succinate, in the medium. Sugars and organic acids were added to 10 mM concentrations, corn oil was added to a concentration of 0.23 mL/L, and casamino acids were added to concentration of 10 g/L. When determining growth inhibition by various antibiotics, cells were cultivated in BB medium supplemented with those listed in Table 1 at 30°C with shaking, and on BB-agar plates incubated at 30°C for 48 h.

**Table 1:** Growth characteristics of *Marinobacter* CP1.

Carbon sources^∗^		Doubling time (h)
Acetate	+	2.6
Lactate	+	1.9
Succinate	+	2.2
Pyruvate	+	1.9
Fumarate		2.8
Casamino acids	+	3.5
Yeast extract/tryptone	+	2.75
Corn oil	+	ND
Glucose	-	NA
Lactose	-	NA
**Electron acceptors^∧^**		
O_2_	++	
Nitrate	+	
Fumarate	-	
Fe(III) citrate	-	
pH range	6–8	
Optimal pH	6.5	
Salinity range	1–14%	
**Antibiotic sensitivity**		
Kanamycin	50 μg/mL	
Gentamycin	10 μg/mL	
Ampicillin	Resistant at 100 μg/mL	
Chloramphenicol	25 μg/mL	
Tetracycline	50 μg/mL on plates, 10–20 μg/mL in liquid	

^∗^*(+) positive for growth, (-) negative for growth. All grown aerobically. ^∧^(++) preferential growth*.

When determining various anaerobic electron acceptors for use by CP1 (Table 1), cells were cultivated in defined modified ASW medium containing 15 mM NaHCO_3_ to increase buffering, instead of 7.38 mM as listed above, and supplemented with 25 mM sodium lactate (NaC_3_H_5_O_3_) as the electron donor at 30°C with shaking, or in ASW supplemented with 5 g/L yeast extract and 10 g/L tryptone at 30°C with shaking. Tubes were prepared in a Coy anaerobic chamber (Coy Lab Products, Grass Lake, MI, United States) with a 10% CO_2_/2.5% H_2_/87.5% N_2_ atmosphere, and sealed with rubber stoppers before being brought out of the chamber for incubation. For iron reduction not linked to growth, cultures were prepared in air and sealed with rubber stoppers, to allow an initial aerobic growth phase.

### Expression of Green Fluorescent Protein in CP1 and Plasmid Stability

Transformation of CP1 was carried out using conjugation by adapting previously published protocols for *Marinobacter* spp. from Bonis and Gralnick (2015), and Sonnenschein et al. (2011). All primers plasmids, and strains used in this study can be found in Table 2. The *E. coli* donor strain WM3064, which is auxotrophic for diaminopimelic acid (DAP), containing pBBR1MCS-2::*gfp*mut3 (Bonis and Gralnick, 2015) was grown in LB supplemented with 0.3 mM DAP and 50 μg/mL kanamycin (Kan) overnight at 37°C with shaking. Following incubation and growth, WM3064 cells were washed once with fresh LB. The CP1 recipient strain was grown in MB for 2 days at 30°C with shaking. Optical densities of cultures were measured and CP1 and WM3064 were mixed in a 3:1 ratio using a volume of 100 μL for the CP1 culture. One hundred microliters of the mixture was then plated onto MB with DAP agar and allowed to soak into the medium. The plate was then incubated for 1 day at 30°C. The resulting conjugation spot was scraped into 1 mL of MB medium, vortexed, spun down, and resuspended in 100 μL of MB, which was spread onto MB agar with Kan (100 μg/mL) and incubated at 30°C for 2 days. After 2 days, multiple GFP-positive colonies were picked and restruck to MB agar with Kan (100 μg/mL) and incubated at 30°C for 2 days. Multiple colonies were picked and restruck again to ensure removal of WM3064 cells. For experiments testing the stability of pBBR1MCS-2::*gfp*mut3 in the absence of antibiotics, CP1 containing pBBR1MCS-2::*gfp*mut3 was inoculated in 5 mL BB liquid cultures at 30°C with shaking. The overnight cultures was diluted 1:100 each day for 10 days in BB broth without selection. After the 10th transfer, the cultures were again diluted and allowed to grow for 6 h before being tested for fluorescence using a C6 accuri flow cytometer (BD Biosciences, San Jose, CA, United States). Aliquotes were also diluted and plated on BB plates with and without Kan to track the loss of the plasmid resistance. As a control, Kan cultures were grown from a plate overnight, then diluted and grown for 6 h before subjecting it to the same tests.

**Table 2:** Primers, plasmids, and strains used in this study.

Primers	Sequence	Source
**dTomato insert**		**This study**
pjQ200skcp1neutF	acgggccagggccagggtaaagatcagcaaagttctagagcggccgccac	This study
pjQ200skcp1neutR	gattttcacagaaaaagaggtatctgacggtctagtggatcccccgggc	This study
CP1neutpJQ200skF	gaattcctgcagcccgggggatccactagaccgtcagatacctctttttc	This study
CP1neutdTomatoR	Tttgcaccattcgatggcgcgccgccatttccctgctattctcaaattagc	This study
lacpromoterCP1neutF	Aaaagccccctgctaatttgagaatagcagggaaatggcggcgcgccatc	This study
lacpromoterdtomatoR	tctttgatgacctcctcgcccttgctcaccatattcacccccgtgaattgactctc	This study
dTomatolacpromoterF	ccggaagagagtcaattcacgggggtgaatatggtgagcaagggcgagg	This study
dTomatoCP1neutR	atttacattaaagtttgatcaaaaaggacccttgtacagctcgtccatgc	This study
CP1neutdTomatoF	ctgtacggcatggacgagctgtacaagggtcctttttgatcaaactttaatgt	This study
CP1neutpjq200skR	agctccaccgcggtggcggccgctctagaactttgctgatctttaccctg	This study
**Wax ester deletion mutants**		
farA-BamHI-a	ATggatccCGAGCGAAAGAATCACCTGG	This study
farA-b	ACCGGGCTCCTGGGTGGGAGAACTCCTTCT	This study
farA-c	AGAAGGAGTTCTCCCACCCAGGAGCCCGGT	This study
farA-SacI-d	ATgagctcCAACTGGCCATCTTCGAACC	This study
acrB-BamHI-a	ATggatccCCAGAACAGTCGCCATGATG	This study
acrB-b	TCGGGGGCAACTGCGCAATCTTCCACCCTG	This study
acrB-c	CAGGGTGGAAGATTGCGCAGTTGCCCCCGA	This study
acrB-SacI-d	ATgagctcGGTCGACTGCTCCTCTTGTA	This study
farA-F503	CCACCTGCTATGTAAACGGC	This study
farA-R683	TTCTTTTCCAGTGCCTTGCC	This study
acrB-F784	CTCAACATCTTCAGCGAGGC	This study
acrB-R978	ACGGGTCGGGTAATTGATGA	This study

**Plasmids**	**Description**	**Source**

pBBR1MCS-2::*gfp*mut3	pBBr1MCS-2 with GFP expressed from constitutive lac promoter	Bonis and Gralnick, 2015
pBBR1MCS-2	Broad range vector with kanamycin resistance and multiple cloning site	Kovach et al., 1995
pJQ200SK	Suicide vector with gentamycin resistance and sacB counter selection	Quandt and Hynes, 1993
pSMV3	Suicide vector with km^R^ and sacB counter selection	Saltikov and Dk, 2003
pJQ200SK::CP1neut-dTomato	Vector for insertion of dTomato in the *Marinobacter* CP1 genome	This study
ZW1205	Vector for farA gene deletion. Uses pSMV3 backbone	This study
ZW1206	Vector for the arcB gene deletion. Uses pSMV3 bakcbone	This study
**Strains**		
*E. coli* UQ950	*E. coli* DH5 (pir) plasmid maintenance strain; F-*(argF-lac)169 80dlacZ58(* M15) *glnV44*(AS) *rfbD1 gyrA96*(NalR) *recA1 endA1 spoT1 thi-1 hsdR17 deoR pir*	Saltikov and Dk, 2003
*E. coli* WM3064	Donor strain for conjugation; *thrB1004 pro thi rpsL hsdS lacZ*ΔM15 *RP4-1360* Δ(*araBAD*)*567* Δ*dapA1341*::[*erm pir*(wt)]	Saltikov and Dk, 2003
*E. coli* DH5α	Plasmid maintenance strain	Woodcock et al., 1989
*Marinobacter* CP1	Wild type *Marinobacter* strain isolated from MCL biofilm	Wang et al., 2015b
CP1 Δ*farA*	*Marinobacter* CP1 with clean delection of *farA*	This study
CP1 Δ*acrB*	*Marinobacter* CP1 with clean delection of *acrB*	This study
CP1 Δ*farA*Δ*acrB*	*Marinobacter* CP1 with clean delection of *farA* and acr*B*	This study
CP1:dTomato	*Marinobacter* CP1 with dTomato under *placI* promoter inserted in the genome	This study

### Insertion of dTomato Into Chromosome

The gene for the fluorescent protein dTomato was inserted into the putative T7 transposon insertion region of CP1 using the suicide plasmid pJQ200sk (Quandt and Hynes, 1993). The intergenic region of the *glm* operon has been shown in other gram negative bacteria to contain a T7 insertion site, and thus be a site in which genomic insertions will not lead to a phenotype due to genomic disruption (Koch et al., 2001; Bose and Newman, 2011). This intergenic region was therefore tested as a site for chromosomal insertions in CP1, at base 1926204. The insertion plasmid was constructed through Gibson cloning (Gibson et al., 2009) and all reagents were purchased from New England Biolabs (Ipswich, MA, United States), including the Q5 polymerase used for PCR reactions. The cloning reaction was transformed into *E. coli* strain DH5α and plated on LB with 10 μg/mL gentamicin plates. The resulting plasmid was purified from a transformed colony, and the insert was sequenced before being transferred to CP1 *via* conjugation as described above except using BB medium instead of MB. The resulting conjugants, which had incorporated the plasmid into the chromosome, were re-struck onto BB gentamicin (10 μg/mL) plates. The resultant strain was then grown in liquid BB broth for 2 days without selection to allow a second recombination event, removing the plasmid, to occur. The liquid culture was then plated on BB agar with 10% sucrose to select for colonies without the SacB counter selection gene contained on pjq200sk. Sucrose selected colonies were screened for dTomato expression, and successful insertion was confirmed through PCR.

### Construction of CP1 In-Frame Deletion Mutants

In-frame deletions of a gene for a short-chain dehydrogenase (NCBI accession number: AKV97721.1) with 91% sequence similarity to the *M. aquaeoli* VT8 fatty *acrB* (NCBI accession number: YP_959769), and the gene for a dehydrogenase (NCBI accession number: AKV97384.1) with 78% similarity to the VT8 *farA* (NCBI accession number: YP_959486) were generated by overlap PCR (Warrens et al., 1997). Briefly, 500 bp DNA fragments upstream and downstream of the target gene open reading frame were amplified from CP1 genomic DNA using two pairs of primers a/b and c/d (Table 2), respectively. The two PCR products were annealed at their overlapping region and amplified using primers a and d. The resulting 1 kb PCR fragment was digested with *Bam*HI and *Sac*I and cloned into the plasmid pSMV3, which contains the Kan resistance gene and the *sac*B gene for counter selection. The resulting gene deletion plasmids, pZW1205 for the *farA* gene homolog and pZW1206 for the *acrB* gene homolog, were transformed into *E. coli* DH5α λ*pir* strain (UQ950) for maintenance. Following growth and plasmid isolation, pZW1205 and pZW1206 were transformed into WM3064 by electroporation. Conjugation was carried out as described above with a few minor changes noted here. In this case, the CP1 recipient strain was grown in MB overnight at 30°C with shaking and 400 μl of each strain was mixed, pelleted, and resuspended in 400 μL LB prior to selection on MB plus Kan (50 μg/mL). The in-frame deletion mutants for the *farA* homolog (Δ*farA*) and *acrB* homolog (Δ*acrB*) were further counter-selected on MB agar plates supplemented with 6% sucrose and verified by PCR with primers a/d and internal primers (Table 2). The double mutant, *farA*Δ*acrB*Δ, was constructed by conjugating pZW1206 plasmid into Δ*farA* mutant.

### Determination of Mutant Phenotype by Gas Chromatography

CP1 was grown in low-calcium ASW plus succinate (26 mM), as well as in the same medium under nitrogen-limiting conditions (0.1 vs. 1 g/L NH_4_Cl) to promote wax ester formation (Barney et al., 2012), by diluting a 3 mL overnight culture at 1:1000 and inoculating into a 48-well plate (500 μL/well). Growth was monitored as increasing optical density at 600 nm every 15 min at 30°C and 258 rpm using a Tecan Infinite M1000 Pro (Grödig, Austria). All measurements were performed in triplicate.

To obtain cell pellets for gas chromatography with flame ionization detector (GC-FID) analysis, 3 mL overnight cultures of CP1, Δ*farA* CP1, Δ*acrB CP1*, and Δ*farA ΔacrB CP1* were grown in triplicate under nitrogen-limiting conditions. Two hundred fifty microliters of the overnight cultures were used to inoculate 250 mL of the same medium and grown for 98 h at 30°C with shaking at 230 rpm. Cells were pelleted at 3500 × *g*, supernatant removed, and pellet resuspended in 8 mL of growth media. Resuspended cells were divided between two 5 mL tubes, centrifuged at 3500 × *g* for 20 min, supernatant removed, and pellets frozen at -80°C.

Samples for GC-FID analysis were prepared and analyzed following the established protocol of Barney and co-workers with some modifications (Gardner et al., 2011; Barney et al., 2012). Briefly, cell pellets were lyophilized (∼0.02 g dry mass), then ground into powder and resuspended in 1 mL 1:1:1 dichloromethane:hexanes:THF. The extraction mixture was vortexed three times at ∼1300 rpm for 10 s, centrifuged at 2000 × *g* for 5 min, and supernatant transferred to a clean vial. This procedure was repeated two additional times using 0.5 mL solvent mixture. To prepare samples for GC-FID measurements, 0.5 mL extraction solution was transferred to a GC sample vial and 5 μL of 10 mg/mL octacosane internal standard was added.

Gas chromatography with flame ionization detector analysis was performed using a Shimadzu QP2010 equipped with a PTV injector and a RTX-Biodiesel TG column (15.0 m × 0.32 mm ID × 0.1 μm df; Restek) with helium as the carrier gas (column flow rate of 0.95 mL/min (total flow rate of 24.7 mL/min) at 19.9 kPa). One microliter of sample was injected at 60°C, and then PTV temperature was increased at 50°C/min to 330°C and held for 15 min. Column temperature was ramped from 60 to 330°C at 10°C/min and held at 330°C for 15 min. Detector temperature was set to 335°C. Varying concentrations (0.05–5 mM) of the wax esters cetyl palmitate and oleyl oleate were used to construct a standard curve (area vs. concentration). Wax ester chain lengths were determined based on comparison with literature values of wax esters analyzed under the same conditions (method and column) and the elution times of cetyl palmitate and oleyl oleate. Because each peak at a given elution time may comprise different wax esters of the same overall chain length, mass of total wax esters was calculated assuming that all wax esters in the sample were saturated linear wax esters. Resulting wax ester concentrations were compared among the different strains using Student’s *t*-test with a two-tailed distribution and homoscedastic variability.

### Imaging

*Marinobacter sp.* CP1 dTomato cells harboring pBBR1MCS-2::*gfp*mut3 were grown in BB medium with 50 μg/mL Kan overnight before adding 1 mL of unfixed cells to a Lab-Tek chambered, single-well #1.0 borosilicate cover glass (Thermo Fisher Scientific, Rochester, NY, United States). Unfixed, attached cells were washed three times with fresh, sterile BB medium with 50 μg/mL Kan before imaging. Fluorescence images were taken on a Nikon A1RSi confocal microscope with a 60× oil immersion and 1.4 numerical aperture objective. GFPmut3 and dTomato fluorescence was excited with the LU-N4 laser unit at 489.4 nm (GFPmut3) and 561.5 nm (dTomato). Emission spectra was collected with a 405/488/461/640 dichroic mirror utilizing 525/50 and 595/50 filters and detected with the GaAsP PMT detector. Images of a single Z plane were collected at the Nyquist limit of a 59.3 μm × 59.3 μm field of view on the NIS Elements software (Nikon Instruments, Melville, NY, United States).

In order to investigate the correlation between OD and cell number in wax ester mutants, samples from 100-h nitrogen limited cultures were fixed with 3.2% paraformaldehyde, then stained with Syto9 DNA stain (Thermo Fisher Scientific, Waltham, MA, United States). After staining, 10 μl of each sample was resuspended in 5 mL ASW, filtered through a 0.2-μm black polycarbonate filter (Millipore, Burlington, MA, United States) and imaged using a Zeiss LSM 800 Airyscan confocal microscope with a Plan-Apochromat 63×/1.4 numerical aperture objective. Syto9 fluorescence was excited at 488 nm using 0.2% laser power, and the emission spectra was collected with 490–562 nm filters at 509 nm detected with an Airyscan detector. Maximum intensity projections of Z-stack images were collected of a 101 μm × 101 μm field of view at 0.46 μm Z-stack intervals with Zeiss Zen Blue imaging software (Carl Zeiss, LLC, Thornwood, NY, United States). The resulting cell counts were compared between strains using a two-tailed, heteroscedastic Student’s *t*-test.

## Results

### Phylogeny and Genome Properties

**FIGURE 1:**
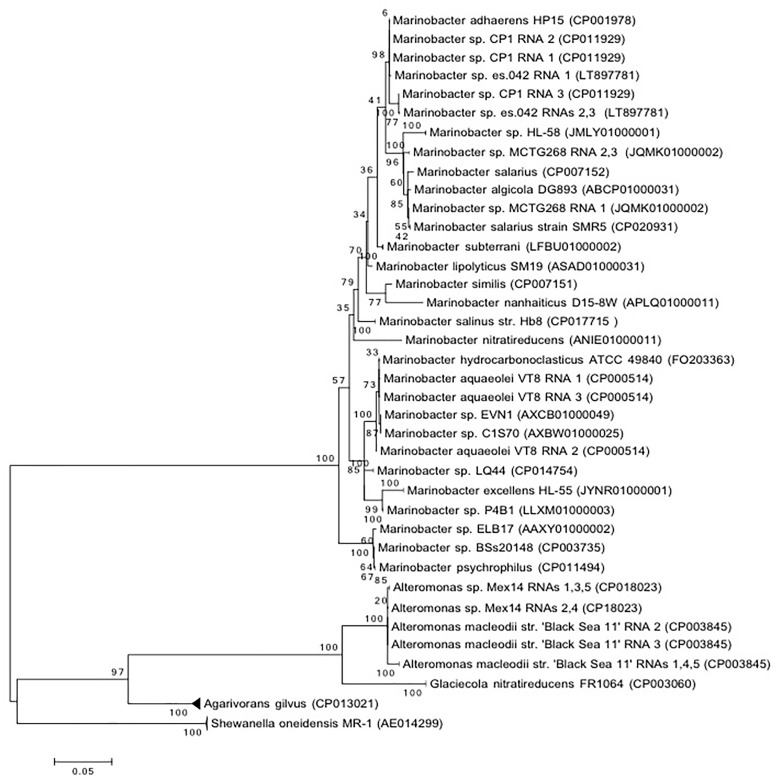
Phylogenetic of CP1 inferred from 16S rRNA sequence. The evolutionary history was inferred by using the maximum likelihood method based on the general time reversible (GTR) model. The percentage of trees in which the associated taxa clustered together is shown next to the branches. If all RNA copies from an organism grouped only with each other, the branch was collapsed. The tree is drawn to scale, with a scale bar showing the number of nucleotide substitutions per site. Sequence source is identified by NCBI accession number.

**FIGURE 2:**
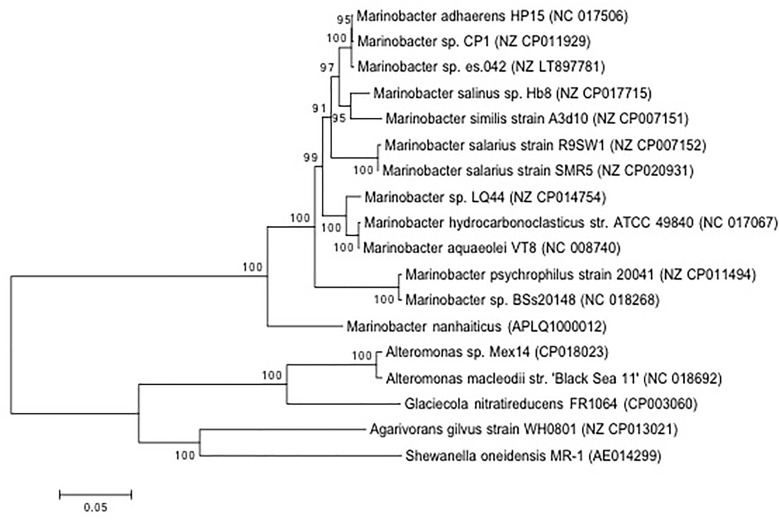
Phylogenetic affiliation of CP1 inferred from proteins from conserved genes. The evolutionary history was inferred by using the maximum likelihood method based on the Le_Gascuel_2008 model. The percentage of trees in which the associated taxa clustered together is shown next to the branches. The tree is drawn to scale, with branch lengths measured in the number of substitutions per site. The analysis involved 18 amino acid sequences. All positions with less than 95% site coverage were eliminated. That is, fewer than 5% alignment gaps, missing data, and ambiguous bases were allowed at any position. Sequence source is identified by NCBI accession number.

Isolation of *Marinobacter* sp. strain CP1 from an autotrophic biocathode community has been previously described (Wang et al., 2015b), and a closed genome for this strain has been published (Wang et al., 2015a). Phylogenetic trees were generated based on 16S and 23S rRNA gene sequences of *Marinobacter* spp. or closely related members of the family *Alteromondaceae* or a selection of core proteins from these species with near complete genomes (Figures 1, 2 and Figure S1 in Supplementary Material). Irrespective of whether the 16S/23S gene or core proteins were used, the closest related organisms to CP1 were *M. adhaerens* HP15 and *Marinobacter* sp. ES.042. While differences in the degree of relatedness to other *Marinobacter* species were noted when using core proteins and whole genome comparison, both 16S and 23S sequences show very little difference across the *Marinobacter* genus. Due to the high degree of identity between the CP1 and HP15 16S and 23S rRNA genes, we further explored whether CP1 should be designated as its own species or represents a strain of *M. adhaerens* using *in silico* genome comparison.

Direct DNA–DNA hybridization (DDH) has traditionally been used to determine whether a strain isolate is a new species (Tindall et al., 2010). In general, if 70% or more of the query DNA hybridizes to the reference genome it is considered the same species and defined as a new strain. An alternative approach is to use *in silico* simulations (dDDH), such as the GGDC, to estimate the probability that two genomes hybridize at or above 70% (Meier-Kolthoff et al., 2013). We used the *in silico* approach to compare CP1 to HP15 and ES.042. This approach resulted in calculated DDH values of 64.2% (CI of 61.2–67%), and 56.6% (CI of 53.9–59.4%), respectively (Table S1 in Supplementary Material). As a control, type strains of *M. salarius*, R9SW1 and SMR5, were hybridized *in silico* and had a calculated DDH value of 80.4% (CI of 77.5–83%), which matches traditional DNA–DNA hybridization approach used to define them as strains. These results indicate that CP1 is a distinct species from *M. adhaerens*. Blastp was used to compare predicted proteins in CP1 and HP15, with a cutoff *e*-value of 10^-6^ and a minimum identity of 20%. Of CP1 proteins, 3658 share at least 20% amino acid identity with at least part of an HP15 protein, 2767 of which have at least 95% amino acid identity. Most of these matches were near full length, although some were only for a small conserved region.

By KEGG pathway annotation, there are 15 pathways that are not duplicated between organisms. These pathways include important metabolic processes, such as the ability to reduce nitrate (described below).

### Growth Conditions

CP1 was originally isolated on MB agar from the Biocathode MCL biofilm (Wang et al., 2015b). Initial transformation and genetic engineering studies were carried out in MB since CP1 was found not to grow in 100% LB. Further optimization of transformation protocols showed that a 50:50 ratio of LB:MB allowed for robust growth and eliminated variability in growth curves observed with 100% MB due to salt precipitation. This medium is referred to as BB (Lysogeny **B**roth and Marine **B**roth) and is currently used for routine growth and cloning. In addition to BB, CP1 is regularly cultivated by our laboratory in defined ASW medium plus succinate (26 mM) as the sole carbon source except where otherwise noted below. Growth curves for BB broth and ASW with succinate are shown in Figure S2 in Supplementary Material. The concentration of CaCl_2_ in the ASW medium is lower than that used to enrich the biocathode community from which CP1 was isolated (0.45 vs. 18 mM) after it was noted that CP1 grew primarily as a surface associated biofilm, rather than planktonically, when grown with 18 mM CaCl_2_.

In order to determine general substrate utilization of CP1, various carbon sources and growth parameters were tested in ASW and the results are summarized in Table 1. CP1 was not able to grow on the sugars glucose or lactose, but was able to utilize organic acids (acetate, lactate, succinate, and pyruvate), amino acids (casamino acids and yeast extract/tryptone), and corn oil as sole carbon sources for growth. CP1 grew preferentially with O_2_ as the electron acceptor vs. anaerobic electron acceptors when lactate was provided as an electron donor. CP1 grew anaerobically in the presence of nitrate in both BB broth and ASW medium with lactate. Anaerobic growth was not observed with fumarate or Fe(III) citrate as the electron acceptor when lactate was provided as the electron donor. However, some ability to reduce Fe(III) citrate relative to an abiotic control was noted in a culture that was permitted to become anaerobic.

Optimal pH and salinity ranges were tested in ASW enriched with 5 g/L yeast extract and 10 g/L tryptone (Table 1). CP1 grew in a pH range between 6 and 8 with optimal growth at 6.5. Growth was similar over a salinity range of 1–14%. CP1 was susceptible to a range of antibiotics including Kan, gentamicin (Gen), chloramphenicol (Chlor), and tetracycline (Tet) (Table 1). Ampicillin (Amp) was partially effective at concentrations greater than 100 μg/mL, which differs from reported ampicillin susceptibility for *M. adhaerens* (Sonnenschein et al., 2011).

### Expression of Green Fluorescent Protein and Plasmid Stability

**FIGURE 3:**
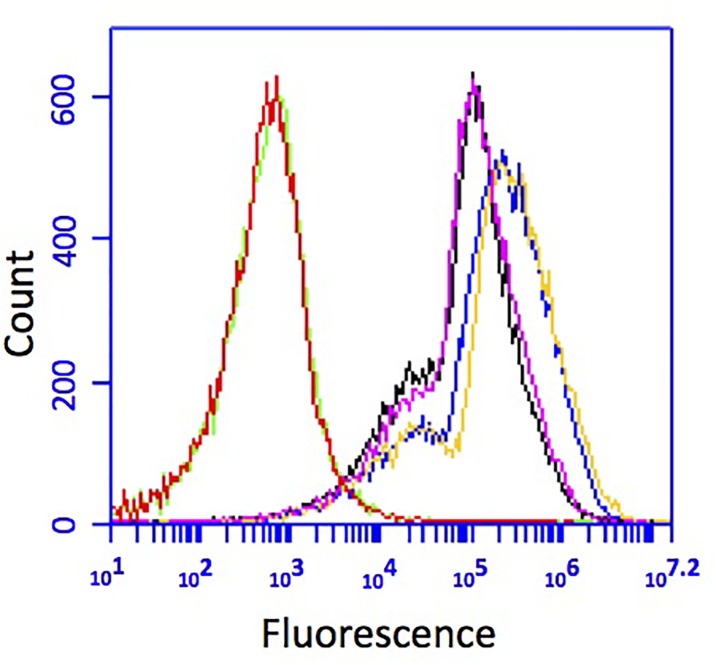
GFP fluorescence of CP1 cultured without kanamycin for 10 transfers. Cells grown and transferred without selection (pink and black) had less fluorescence than cells grown with kanamycin (yellow and blue), but both were above the background fluorescence of wild-type cells (red and green).

Transformation of CP1 with broad host range vector pBBR1MCS-2::*gfp*mut3 containing green fluorescent protein (GFP) was attempted *via* conjugation, chemical transformation, and electroporation. Conjugation with the *E. coli* donor strain WM3064 was successful, whereas chemical transformation and electroporation were not. To test the stability of pBBR1MCS-2::*gfp*mut3 over time in the absence of antibiotic selection, CP1 was serially passaged without kanamycin (Kan-) over 10 days and flow cytometry and plate counts with and without selection recorded. The cultures passaged without selection showed a slight decrease in peak fluorescence by flow cytometry, but still well above the background fluorescence of wild type (Figure 3) The plate counts of the cultures passaged without Kan showed 72% of the colonies counted on non-selected medium, vs. 86% in cultures grown with Kan. This indicates that CP1 maintains the plasmid even in the absence of antibiotic selection, although there may be some loss over time. In addition, the pBBR1MCS-2::*gfp*mut3 was recovered from Kan+ cultures by plasmid prep and no change to the nucleotide sequence was observed by resequencing (data not shown).

**FIGURE 4:**
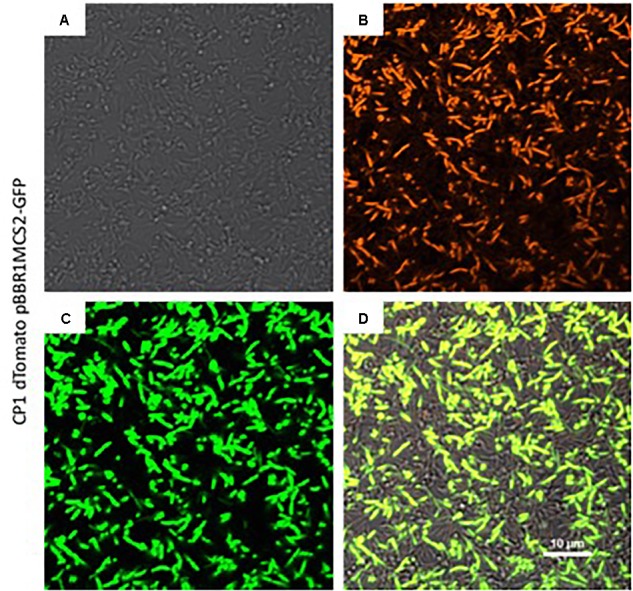
Fluorescence imaging of expression of green fluorescent protein (GFP) from the pBBR1MCS-2::*gfp*mut3 construct in the dTomato background strain of *Marinobacter* sp. CP1. **(A)** Transmitted light image of cells grown in BB medium in a chambered coverglass. **(B)** Same cells as **(A)**, except at excitation of 561 nm to visualize dTomato, and **(C)** at excitation of 488 nm to visualize GFP. **(D)** Merged image of the 561 and 488 channels.

In order to create a fluorescent background strain of CP1, the orange fluorescent protein, dTomato (Shaner et al., 2004), was cloned into the T7 transposon insertion region of CP1 which is considered a neutral site, i.e., not likely to cause disruption in expression patterns (Koch et al., 2001) under the control of the constitutive LacI promoter. The resulting strain expressed dTomato during growth, detectable on a fluorescence plate reader by an increase in fluorescence at 581 nm with an excitation of 554 nm, and visible by fluorescence microscopy (Figure 4). Additionally, the pBBR1MCS-2::*gfp*mut3 plasmid was transformed into the CP1 dTomato strain to demonstrate the utility of this strain to co-express fluorescent reporters (Figure 4). The expression of multiple fluorescent proteins allows for future imaging studies, and demonstrates the ability of the organisms to handle multiple synthetic constructs, an important capability for future synthetic biology applications.

### Double Mutant in a Putative Wax Ester Synthesis Pathway Results in Loss of Wax Ester Production

**FIGURE 5:**
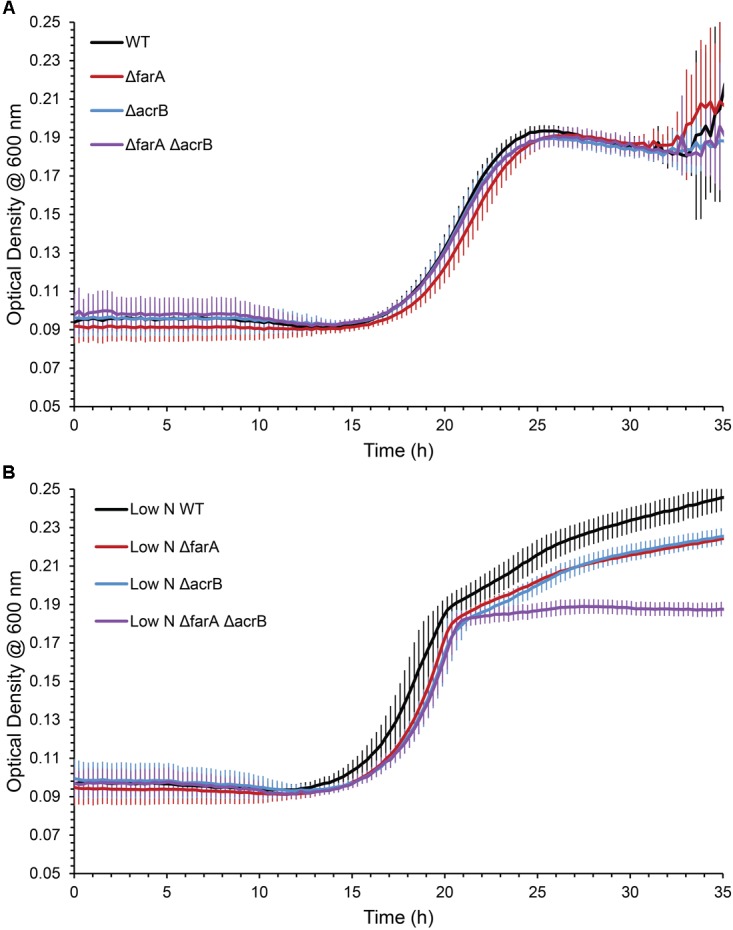
Average (*n* = 3) OD_600nm_ of WT, Δ*farA*, Δ*acrB*, and Δ*farA ΔacrB* strains of CP1 under **(A)** regular growth conditions (ASW media, 26 mM succinate) and **(B)** low nitrogen growth conditions (ASW, 26 mM succinate, 0.1 g/L NH_4_Cl) for 35 h.

**FIGURE 6:**
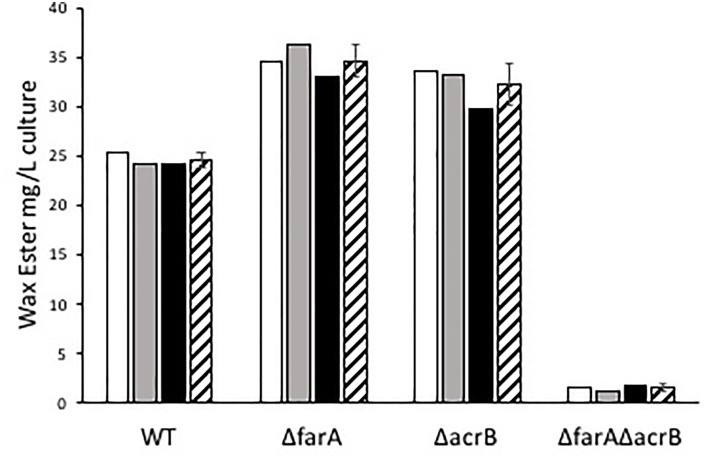
Mg of wax esters per liter of WT, Δ*farA*, Δ*acrB*, and Δ*farA*Δ*acrB Marinobacter* sp. CP1 produced under nitrogen-limited growth conditions. White, gray, and black bars show individual samples, while the striped bar shows the average. Error bars denote standard deviation (*n* = 3). Mass of wax esters was calculated assuming all wax esters are saturated linear wax esters.

*Marinobacter* spp. are known to produce wax esters (Barney et al., 2012; Bertram et al., 2017), and enzymes of this pathway have been used for biotechnology applications (Miklaszewska et al., 2018). For example, Liu et al. (2013) used *acrB* from *M. aquaeolei* VT8 to produce fatty alcohols in an engineered strain of *E. coli*. Here, we investigated whether CP1 has a similar wax ester synthesis pathway to that described for *M. aquaeolei* VT8. Genes for a short-chain dehydrogenase (AKV97721.1) and dehydrogenase (AKV97384.1) found to have 91 and 78% nucleotide similarity to a *farA* and *acrB*, from VT8 were deleted (Lenneman et al., 2013). When grown in typical ASW with succinate (26 mM), WT and single or double mutant strains of the *farA* (Δ*farA*) and *acrB* (Δ*acrB*) homologs showed no differences in growth (Figure 5). However, when the nitrogen concentration was reduced to that reported to induce wax ester synthesis (0.1 g per liter vs. 1 g) (Lenneman et al., 2013), a biphasic growth curve was observed by OD after 20 h for both the WT strain and, to a lesser extent the single knockouts, but not for the double deletion strain (Δ*farA*Δ*acrB*). To confirm that the biphasic growth feature was due to a loss of wax ester synthesis, GC-FID analysis of cell pellets from cultures grown under nitrogen-limiting conditions for 100 h (Figure S3 in Supplementary Material) was performed (Figure 6). Additionally, cell counts were performed at 100 h to determine whether the higher OD correlated to higher cell numbers. The results (Figure S3C in Supplementary Material) showed that the double mutant had an average of 1.5 × 10^8^ cells/mL while the wild type had 4.9 × 10^8^ cells/mL, which was the same order of magnitude and was not significantly different by Student’s *t*-test (*p* > 0.05). Peak retention times associated with wax esters were the same among all strains (Figure S4 in Supplementary Material); however, the total peak area was dramatically reduced for the Δ*farA*Δ*acrB* strain compared to both the wild-type and single knockout strains. Interestingly, the total wax esters measured for both single knockout strains was significantly higher than the wild type strain (determined using Student’s *t*-test: *p*-value for wild type vs. Δfar = 0.00057; *p*-value for wild type vs. ΔarcB = 0.00424). A small amount of wax esters were detected in the cell pellets of the double mutant, consistent with previous measurements of the *M. aquaeoli ΔfarAΔacrB* double mutant (Lenneman et al., 2013).

## Discussion

Phylogenetic comparison of *Marinobacter* sp. CP1 to other *Marinobacter* spp. and closely related *Alteromonadaceae* revealed that CP1 is most closely related to *M. adhaerens* HP15. As noted above, *in silico* comparison of CP1 to HP15 resulted in a calculated dDDH value of 64%, which is below the suggested 70% cutoff traditionally used for experimental DDH to define a new species. Therefore, we propose the new species *Marinobacter atlanticus* sp. nov. (at.lan.ti’cus. L. masc. adj. *atlanticus* of the Atlantic ocean).

Optimal growth conditions for *M. atlanticus* CP1, including salinity range and carbon utilization, were similar to those reported for other *Marinobacter* spp. isolated from the marine environment (Handley and Lloyd, 2013). The optimal growth pH of 6.5 was slightly lower than that of other species, including *M. adhaerens* which has been reported to prefer a more basic pH (7.0–8.5). *M. adhaerens* can also tolerate more saline conditions (0.5–20%) (Kaeppel et al., 2012) than *M. atlanticus*. CP1 is susceptible to common antibiotic growth inhibitors, with the exception of ampicillin, which may be useful for implementation of various transformation vectors for future genetic studies. We observed that the concentration of CaCl_2_ influences whether cells grow more in an aggregated or planktonic state. Calcium concentration may be one factor contributing to more robust planktonic growth in BB medium, which has a CaCl_2_ concentration of 8 mM, as opposed to MB which has 16 mM CaCl_2_. While not previously reported for *Marinobacter* spp., members of the related family *Pseudoalteromonadaceae* experience the reverse effect, where low concentrations of calcium induce biofilm formation, possibly through regulation by cyclic di-GMP (Patrauchan et al., 2005). Biofilm formation can be modulated by a number of chemical and biological signaling events and further investigation of this phenomenon is underway for CP1.

CP1 was not able to grow using Fe(III) citrate as the sole electron acceptor; however, a small amount of iron was reduced (*ca.* 120 μM) in culture tubes that were sealed and allowed to become anaerobic. This is consistent with previous observations that *Marinobacter* spp. easily shift metabolic activity in the presence of O_2_ gradients (Handley and Lloyd, 2013). The ability of *Marinobacter* spp. to reduce/oxidize metals without requiring them for growth could point to a larger role in biogeochemical cycling, as suggested by Singer et al. (2011) and Handley and Lloyd (2013). Although the closely related *M. adhaerens* cannot reduce nitrate (Kaeppel et al., 2012), CP1 was able to utilize nitrate as an electron acceptor for anaerobic growth, presumably to nitrite. In 2013, Handley and Lloyd (2013) reported nitrate reduction in 24 out of 31 species that had been tested, eight of which were respiratory, indicating that it is likely a common trait for this genus. Evans et al. (2018) has suggested that denitrification pathways in *Marinobacter* species may have biogeochemical implications in the subsurface. The respiratory nitrate reduction pathway for *M. hydrocarbonoclasticus* has been described (Marangon et al., 2012) and can also be found in CP1. It is conceivable that it is active during anaerobic growth although we did not specifically explore that question here.

Wax ester synthesis occurs in *M. aquaeolei* VT8 under nitrogen-limiting conditions, and it has been shown that simultaneous deletion of genes for both the fatty *acrB* and *farA* results in a dramatic loss in production of wax esters (Lenneman et al., 2013). Here, deletion strains for CP1 *acrB* and *farA* gene homologs were generated and a phenotype was observed for both growth and wax ester synthesis under nitrogen-limiting conditions. Deletion of either gene individually resulted in a statistically significant *increase* in the total wax esters recovered from cell pellets. These results differ from *M. aquaeolei* VT8 where the wax ester production in the Δ*farA* deletion strain was greatly diminished while wax ester production in the Δ*acrB* deletion strain was the same as wild type (Lenneman et al., 2013). Deletion of both genes simultaneously appears to have nearly eliminated wax ester production, consistent with the behavior observed for Δ*farA*Δ*acrB* VT8. Similar to VT8, CP1 produces wax esters estimated to range from C_28_ to C_36_ based on retention times (Barney et al., 2012). However, in CP1, C_32_ length wax esters were the most abundant compared to VT8 in which the majority were C_34_ wax esters. Wild type *M. aquaeoli* VT8 has been reported to produce *ca.* 7 mg/0.2 g dry cell pellet, while by our estimate, *M. atlanticus* CP1 wild type produced *ca.* 35 mg total wax ester/g dry cell pellet and single deletion strains of either *acrB* or *farA* produced *ca.* 150 mg total wax ester/g dry cell pellet, warranting further investigation of these enzymes for biotechnology applications.

## Conclusion

*Marinobacter atlanticus* CP1 is a genetically tractable, heterotrophic marine bacterium isolated from the Biocathode MCL electrode community. Its growth characteristics are similar to many other species of *Marinobacter* isolates reported thus far, including optimal pH, salinity, and carbon utilization. In addition, *M. atlanticus* is able to use nitrate as a terminal electron acceptor for anaerobic growth where the closely related *M. adhaerens* HP15 is not. *M. atlanticus* is able to produce wax esters under nitrogen-limiting conditions using a pathway with homology to the model organism *M. aquaeolei* VT8. Genetic tools for CP1 developed in this study will be critical to understanding the role of this organism in cathodic current generation in Biocathode MCL, as well as expanding our understanding of wax ester synthesis in the *Marinobacter* genus.

## Author Contributions

BC and JD tested growth substrates and conditions. LB and ZW developed the genetic system. AM and BE performed phylogenetic analysis and interpretation. EO, BJ, and MM performed wax ester experiments. DP performed microscopy and imaging. SG devised experiments and wrote the manuscript.

## Conflict of Interest Statement

The authors declare that the research was conducted in the absence of any commercial or financial relationships that could be construed as a potential conflict of interest.

## Footnotes

1 https://img.jgi.doe.gov/

2 http://www.ncbi.nlm.nih.gov/

3 http://ggdc.dsmz.de/ggdc.php\#
