# Supplementary material for: Development of a Genetic System for Marinobacter atlanticus CP1 (sp. nov.), a Wax Ester Producing Strain Isolated From an Autotrophic Biocathode
Source: Front Microbiol. 2018 Dec 21;9:3176. doi: 10.3389/fmicb.2018.03176 (PMC6308636; doi:10.3389/fmicb.2018.03176)
Supplement: Supplementary file 2 [file Table_2.docx]

**Supplemental Materials**

**Supplemental Tables**

S1. GGDC results

Will be uploaded as a separate Xcel sheet.

**Supplemental Figures**


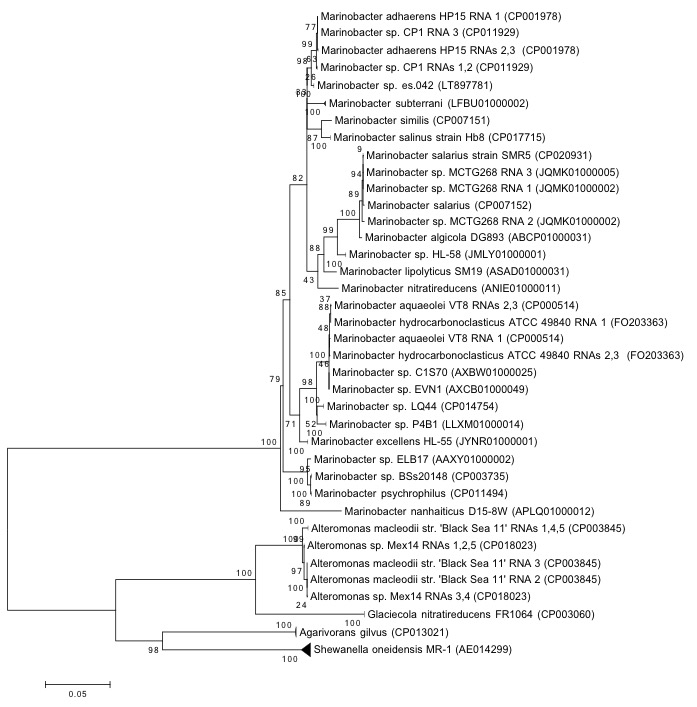


S1**.** Phylogenetic affiliation of CP1 inferred from 23S rRNA sequence. The evolutionary history was inferred by using the Maximum Likelihood method based on the General Time Reversible (GTR) model. The percentage of trees in which the associated taxa clustered together is shown next to the branches. If all RNA copies from an organism grouped only with each other, the branch was collapsed. The tree is drawn to scale, with a scale bar showing the number of nucleotide substitutions per site. Sequence source is identified by NCBI accession number.

S2. Optical density of wild type CP1 in BB broth and low calcium artificial seawater (ASW) with 26 mM succinate over time measured by absorbance at 600 nm. Bars represent the standard deviation of 3 replicates.


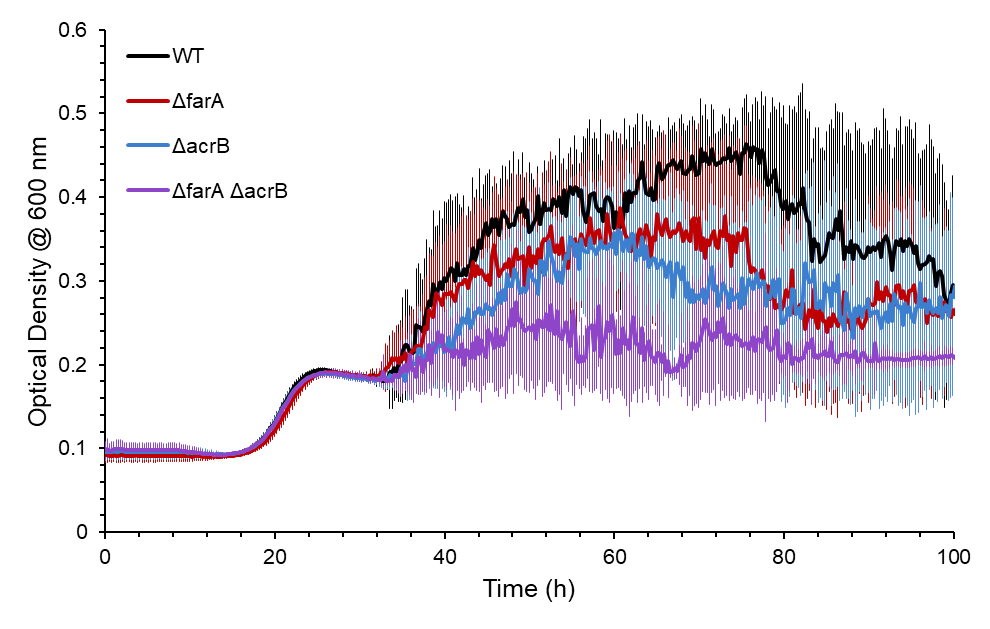


B.

A.


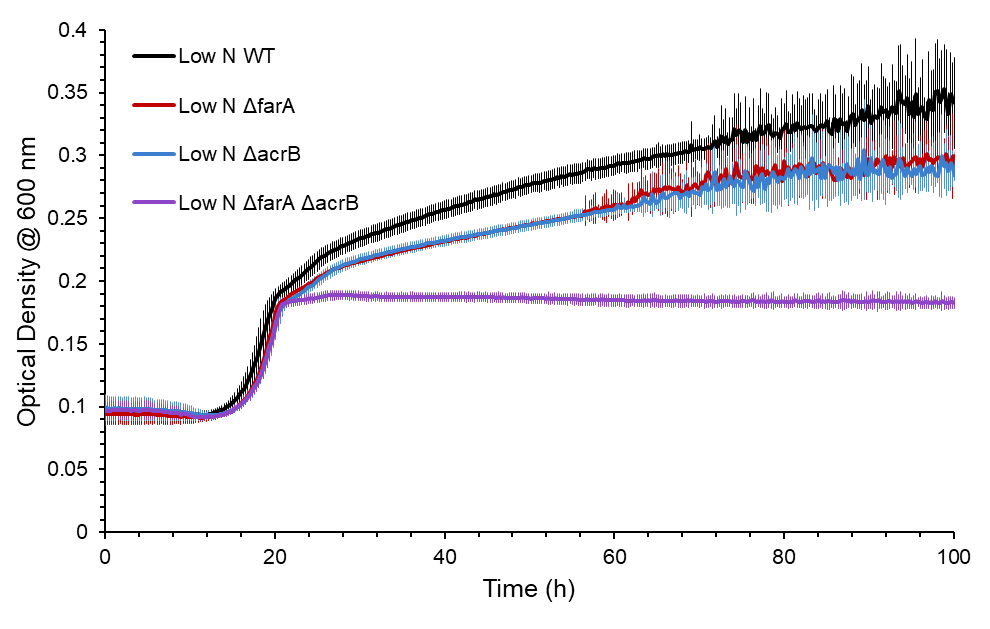


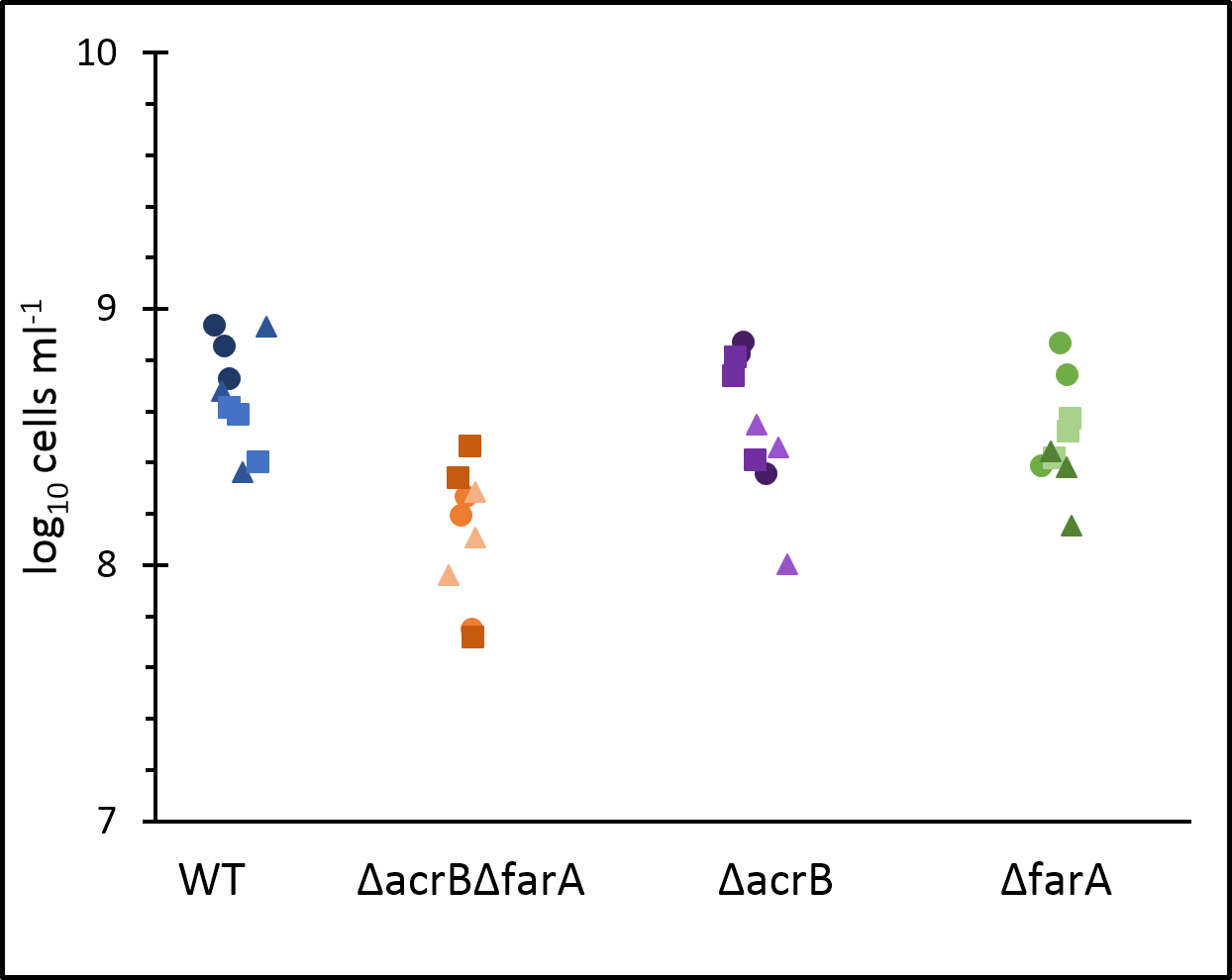


C.

S3. Average (n=3) OD_600nm_ of WT, *ΔfarA*, *ΔacrB*, and *ΔfarA ΔacrB* strains of CP1 under A) regular growth conditions (ASW media, 26 mM succinate) and B) low nitrogen growth conditions (ASW, 26 mM succinate, 0.1 g/L NH_4_Cl) for 100 hours. These samples were used for wax ester analysis. Bars represent the standard deviation. C) cell counts at 100 hours under low nitrogen conditions. Circles, squares, and triangles represent biological replicates.


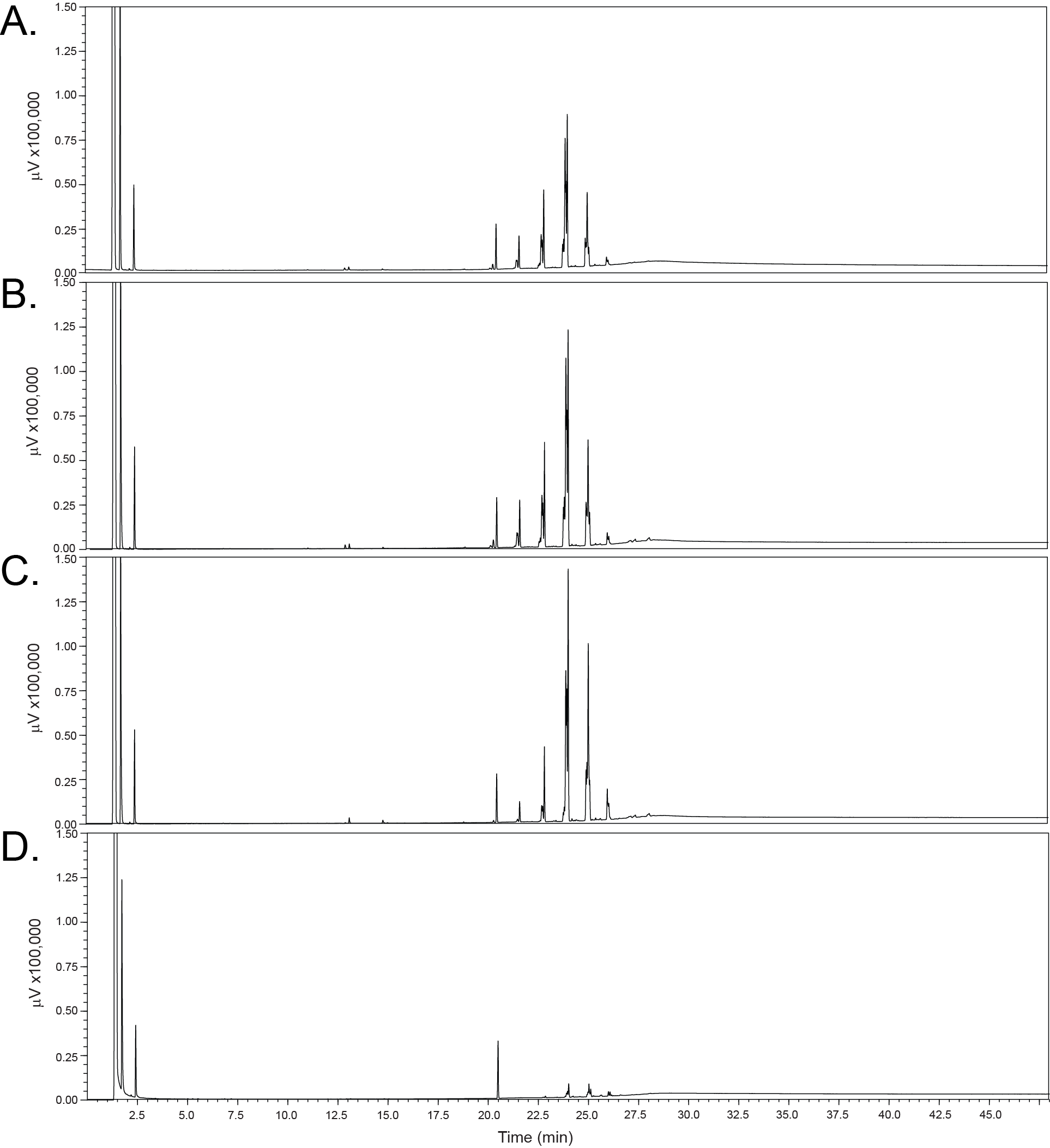


S4. GC-FID chromatograms solvent-extracted cell pellets from A) *M. atlanticus*, B) ΔfarA *M. atlanticus*, C) ΔacrB *M. atlanticus*, and D) ΔfarAΔacrB *M. atlanticus* grown under nitrogen-limiting conditions. The peak seen at 20.5 min is the internal standard, octacosane.
